# The Role of Patient‐ and Drug‐Related Factors in Oral Minoxidil and Pericardial Effusion: Analyses of Data From the United States Food and Drug Administration Adverse Event Reporting System

**DOI:** 10.1111/jocd.16732

**Published:** 2024-12-16

**Authors:** Aditya K. Gupta, Mary A. Bamimore, Robert Haber, Greg Williams, Vincent Piguet, Mesbah Talukder

**Affiliations:** ^1^ Mediprobe Research Inc. London Ontario Canada; ^2^ Division of Dermatology, Department of Medicine, Temerty Faculty of Medicine University of Toronto School of Medicine Toronto Ontario Canada; ^3^ Department of Dermatology Case Western Reserve University School of Medicine Cleveland Ohio USA; ^4^ Farjo Hair Institute London UK; ^5^ Division of Dermatology Women's College Hospital Toronto Ontario Canada; ^6^ School of Pharmacy BRAC University Dhaka Bangladesh

**Keywords:** adverse effect, alopecia, androgenetic alopecia

## Abstract

**Background:**

While oral minoxidil (OM) has been associated with pericardial effusion (PE), its etiology is presently inconclusive.

**Aims:**

We characterized patient‐ and drug‐related factors across reports from the United States Food and Drug Administration Adverse Event Reporting System (FAERS) for PE and OM.

**Methods:**

Our observation period spanned 18.5 years. Parametric and non‐parametric analyses were used; we stratified our findings according to two groups of adverse events (AEs), namely, PE and all other AEs.

**Results:**

Across reports of OM (*n* = 2747), positive dechallenge (complete resolution or subsiding of AE upon discontinuation of OM) was significantly more likely to occur for PE than for all other AEs (*p* < 0.05). Furthermore, OM was significantly more likely to play a primary role in PE compared to all other AEs (*p* < 0.05). The proportion of men was significantly higher in OM reports of PE than in OM reports of all other AEs (*p* < 0.05). We also identified six reports of PE and topical minoxidil.

**Conclusions:**

Though findings from spontaneously reported data never prove causality, our findings on dechallenge and purported role may suggest one. There were no reports of PE at a dose < 2.5 mg/day, 2/35 reports at 2.5 mg/day, and 8/35 reports at 5 mg/day. Overall, the results of statistical analyses support that the relationship between OM and PE is dose independent. Caution should also be taken when applying minoxidil topically because of reports of PE associated with this route of administration.

## 1 Introduction

Oral minoxidil has recently been used, off‐label, for the management of androgenetic alopecia. However, its use is also synonymous with adverse events, including pericardial effusion [1]. This adverse event has been cited in many published case reports and retrospective studies [1, 2, 3, 4, 5]. Pericardial effusion occurs when excess fluid accumulates in the pericardial sac surrounding the heart. This fluid buildup can increase pressure on the heart, potentially hindering its proper function. Predisposing factors include inflammation, cancer, trauma, and sometimes no identifiable cause, known as idiopathic pericarditis [6].

Oral minoxidil is a vasodilator; inceptively, the agent's indicated use was for hypertension [7]. According to a recent meta‐analysis, oral minoxidil and topical minoxidil solutions have similar efficacy and safety profiles in the management of androgenetic alopecia [8]. However, many consumers find the systematic form of minoxidil preferable to the topical/transdermal form for various reasons including the fact that oral administration is less cumbersome (i.e., more convenient) than topical application [9].

In the current study, we examined reports from the United States Food and Drug Administration Adverse Event Reporting System (FAERS) to characterize and analyze this adverse event's relationship with the vasodilator. Hitherto, the literature on oral minoxidil's etiology in pericardial effusion has been inconclusive; whether or not the etiological role is causal remains unresolved. So, our work attempted to address some knowledge gaps.

## 2 Methods

Our work was conducted in accordance with *STrengthening the Reporting of OBservational studies in Epidemiology* (STROBE) [10] recommendations. Though we used spontaneously reported data from FAERS, the current study did not necessitate guidance from READUS‐PV (i.e., *REporting of A Disproportionality analysis for drUg Safety signal detection using individual case safety reports in PharmacoVigilance*) [11] as the current study did not perform any disproportionality analyses.

Our observation period spanned 18.5 years as we used data spanning first quarter of 2006 to the second quarter of 2024. The FAERS comprise data that are housed across seven files and published on a quarterly basis. The current study used information from the *DEMO*, *REAC*, *DRUG*, and *INDI* files.

The current study characterized patient‐ and drug‐related characteristics across reports of pericardial effusion and oral minoxidil; information pertaining to age, sex, and weight were obtained from the *DEMO* dataset; adverse events are detailed in the *REAC* file. Indication for use is in *the INDI* file; information regarding a drug's purported role and total daily dose was obtained from the *DRUG* file.

The drug‐related information we analyzed included details of dechallenge and purported role. In the FAERS, a dechallenge is positive if the adverse event is abated (i.e., disappears or subsides) after drug use is halted; a negative dechallenge occurs when the adverse event persists even after drug use is halted. In terms of purported role, the primary role alludes to the drug being the primary suspect in the occurrence of the AE; the secondary role refers to the drug being deemed a secondary suspect in the occurrence of the untoward effect. Similarly, “interaction” role alludes to the AE being a result of some drug interaction; concomitant corresponds to the drug co‐existing.

Wherever applicable, we conducted nonparametric and parametric tests; all statistical analyses were conducted with R software [12]. In all analyses, alpha (i.e., cut‐off for statistical significance) was set to 5%.

## 3 Results

Across the observation period of 18.5 years, there were 2747 reports for orally administered minoxidil where the reports had complete information on the total daily dose (up to 100 mg daily); of these reports, 35 pertained to the occurrence of pericardial effusion. All statistical analyses were based on the 2747 reports (PE = 35, No PE = 2712).

### 3.1 Dose

**FIGURE 1:**
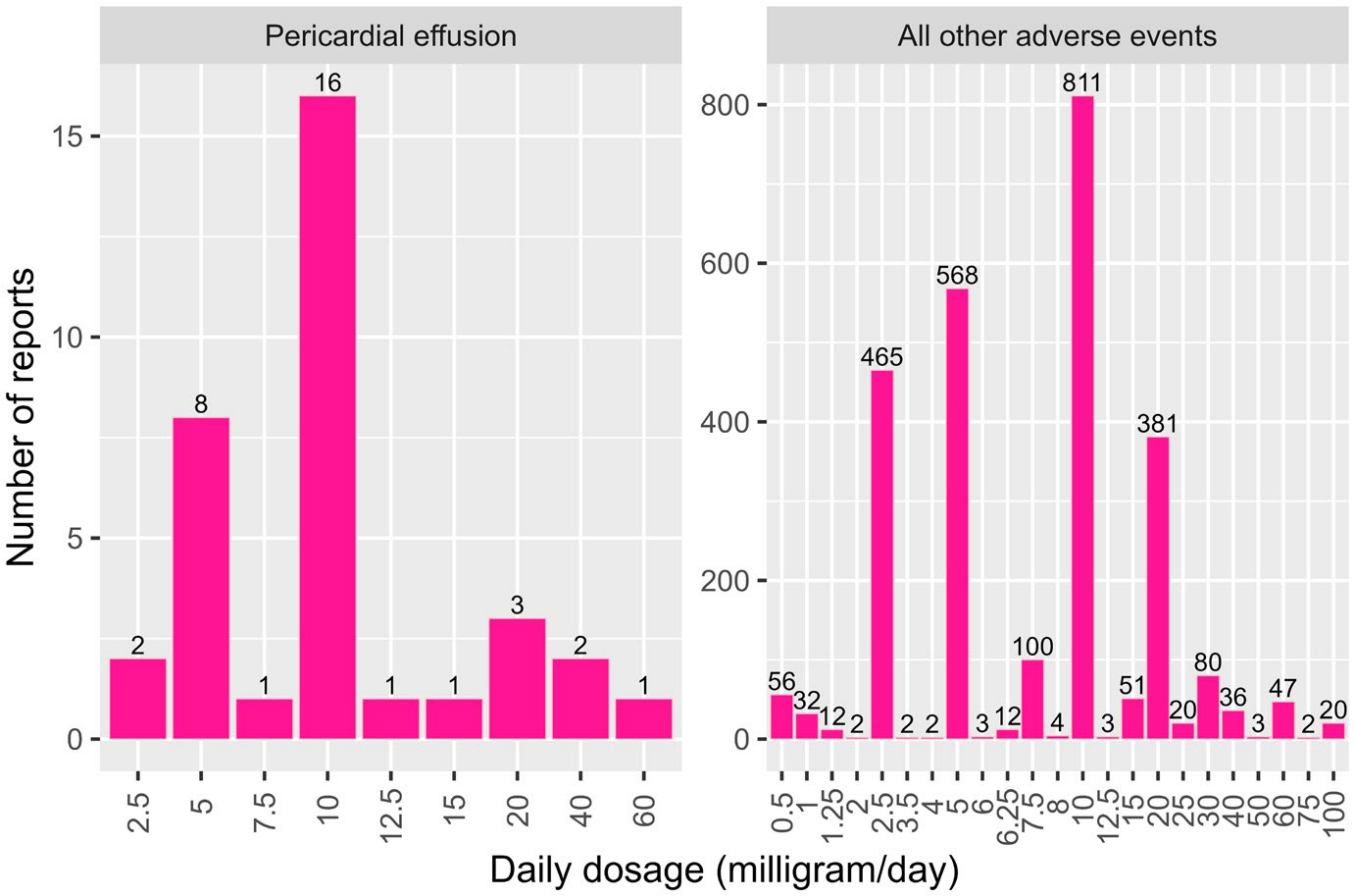
Visual presentation for the distribution of daily dose across reports of oral minoxidil (equal to or under 100 mg per day). There were 35 pericardial effusion reports, while all other adverse event reports totaled 2712.

Across the reports, there was a finite number of total daily doses for oral minoxidil, 23 distinct doses ranging from 0.5 mg daily to 100 mg daily. We examined the relationship between total daily dose of oral minoxidil and the occurrence of pericardial effusion. In the occurrence of thereof, the most common total daily dose reported was 10 mg daily; similarly, across all other adverse events, the most common dose was also 10 mg daily (Table 1). The distribution was stratified by the occurrence of pericardial effusion versus all other adverse events, and the visual depiction of this distribution—as per the two strata are presented in Figure 1. Pearson's correlation showed no significant relationship between dose and adverse events as per the two‐sided (*p* = 0.60) and one‐sided (*p* = 0.30) p‐values. Kendall's correlation also showed nonsignificant relationship (two‐sided *p* = 0.13, one‐sided *p* = 0.065).

**TABLE 1:** Summary of information on total daily dose across reports—from the United States Food and Drug Administration Adverse Event Reporting System (FAERS)—pertaining to oral minoxidil users in whom pericardial effusion did and did not occur between 1st quarter of 2006 and the 2nd quarter of 2024.

Characteristic	Reports of pericardial effusion *N* = 35^a^	Reports of all other adverse events *N* = 2712^a^
Daily dosage (mg/day)		
0.5	0/35 (0%)	56/2712 (2.1%)
1	0/35 (0%)	32/2712 (1.2%)
1.25	0/35 (0%)	12/2712 (0.4%)
2	0/35 (0%)	2/2712 (< 0.1%)
2.5	2/35 (5.7%)	465/2712 (17%)
3.5	0/35 (0%)	2/2712 (< 0.1%)
4	0/35 (0%)	2/2712 (< 0.1%)
5	8/35 (23%)	568/2712 (21%)
6	0/35 (0%)	3/2712 (0.1%)
6.25	0/35 (0%)	12/2712 (0.4%)
7.5	1/35 (2.9%)	100/2712 (3.7%)
8	0/35 (0%)	4/2712 (0.1%)
10	16/35 (46%)	811/2712 (30%)
12.5	1/35 (2.9%)	3/2712 (0.1%)
15	1/35 (2.9%)	51/2712 (1.9%)
20	3/35 (8.6%)	381/2712 (14%)
25	0/35 (0%)	20/2712 (0.7%)
30	0/35 (0%)	80/2712 (2.9%)
40	2/35 (5.7%)	36/2712 (1.3%)
50	0/35 (0%)	3/2712 (0.1%)
60	1 / 35 (2.9%)	47/2712 (1.7%)
75	0/35 (0%)	2/2712 (< 0.1%)
100	0/35 (0%)	20/2712 (0.7%)

*Note:* This summary is based on observations (i.e., reports) where (a) total daily dose information was available and (b) the route of administration was deemed oral (*n* = 2747). Statistical analyses (including generalized linear regressions (GLM)) were based on this set of observations.

^a^ *n*/*N* (%).

The Wilcoxon rank sum test showed that there was no significant difference between the distribution of the total daily dose of oral minoxidil between reports of PE and all other AEs (*p* = 0.13). Furthermore, there was no significant correlation between dose and occurrence of pericardial effusion as per Pearson's correlation (*p* = 0.60). In this dataset, there were no records of pericardial effusion occurring at a dose of less than 2.5 mg/day. There were 2/35 reports at 2.5 mg/day and 8/35 reports at 5 mg/day (Table 1).

### 3.2 Indication for Use

Hypertension was the most common “indication for use” in reports for both pericardial effusion and all other adverse events. Furthermore, this indication was significantly higher (*χ* ^2^ = 4.8364, *p* = 0.03) in reports for pericardial effusion than for all other adverse events. For many reports, the indication was unknown. Of the reports on oral minoxidil use and pericardial effusion, hair loss‐related conditions were an indication for use in 6.7% of these. Across the reports of oral minoxidil use and all other adverse events, hair loss‐related conditions were an indication for use in 4.4% of these. There was no significant difference between the proportion of reports for which hair loss‐related condition was an indication for use (*χ* ^2^ = 0.017, *p* = 0.90).

### 3.3 Age, Weight, and Sex

The mean age of oral minoxidil users across reports of pericardial effusion (60.5 ± 15.9 years) and all other adverse events (57.4 ± 17.1 years) (Table 2) was not significantly different according to both Welch two‐sample *t*‐test (*p =* 0.25), and Wilcoxon rank sum test (*p* = 0.48).

**TABLE 2:** Summary of report characteristics—from the United States Food and Drug Administration Adverse Event Reporting System (FAERS)—pertaining to oral minoxidil users in whom pericardial effusion did and did not occur between 1st quarter of 2006 and the 2nd quarter of 2024.

Characteristic	Reports of pericardial effusion *N* = 35^a^	Reports of all other adverse events *N* = 2712^a^
Age, years	60.50 (15.19)	57.44 (17.05)
(Missing)	1	526
Sex		
Female	4/35 (11%)	896/2529 (35%)
Male	31/35 (89%)	1633/2529 (65%)
(Missing)	0	183
Weight, kilograms	88.17 (28.12)	85.90 (24.67)
(Missing)	6	1001
Dechallenge		
Positive dechallenge	14/21 (67%)	181/542 (33%)
Negative dechallenge	1/21 (4.8%)	78/542 (14%)
Does not apply	2/21 (9.5%)	122/542 (23%)
Unknown	4/21 (19%)	161/542 (30%)
(Missing)	14	2170
Rechallenge		
Positive rechallenge	0/16 (0%)	31/860 (3.6%)
Negative rechallenge	0/16 (0%)	4/860 (0.5%)
Does not apply	14/16 (88%)	650/860 (76%)
Unknown	2/16 (13%)	175/860 (20%)
(Missing)	19	1852
Purported role of drug		
Primary Suspect Drug	24/35 (69%)	427/2712 (16%)
Secondary Suspect Drug	6/35 (17%)	189/2712 (7.0%)
Interacting	0/35 (0%)	6/2712 (0.2%)
Concomitant	5/35 (14%)	2090/2712 (77%)
Reporter's occupation		
Physician	4/34 (12%)	546/2480 (22%)
Pharmacist	19/34 (56%)	286/2480 (12%)
Other Health Professional	10/34 (29%)	634/2480 (26%)
Consumer	1/34 (2.9%)	914/2480 (37%)
Lawyer	0/34 (0%)	100/2480 (4.0%)
(Missing)	1	232

*Note:* This summary is based on observations (i.e., reports) where (a) total daily dose information was available and (b) the route of administration was deemed oral (*n* = 2747). Statistical analyses (including generalized linear regressions [GLM]) were based on this set of observations.

^a^ Mean (SD); *n*/*N* (%).

The mean weight across reports of pericardial effusion (88.2 kg ± 28.1 kg) and all other adverse events (85.9 kg ± 24.7 kg) (Table 2) was not significantly different according to both Welch two‐sample t‐test (*p* = 0.67), and Wilcoxon rank sum test (*p* = 0.76).

The proportion of male oral minoxidil users across reports of pericardial effusion (89%) was significantly higher than that across reports of all other AEs (65%) (*χ* ^2^ = 7.7075, *p* = 0.005).

### 3.4 Purported Role

Across our entire sample of observations (i.e., 2747 reports), oral minoxidil's purported role in the occurrence of any adverse event was either primary, secondary, concomitant, or interacting. Across reports for all other adverse events, oral minoxidil's purported role was mostly concomitant (77%). The purported role was primary in 16% of reports—while this agent's purported role was primary across 69% of reports for pericardial effusion (Table 2); furthermore, this difference in the distribution of proportions was statistically significant according to Fisher's Exact Test (*p* < 0.001).

### 3.5 Dechallenge

While positive dechallenge was reported in 33% of reports for all other adverse events, it was reported in a significantly greater percentage (67%) of reports for pericardial effusion (*χ* ^2^ = 8.4705, *p* = 0.0036) (Table 2).

## 4 Discussion

The story between oral minoxidil and pericardial effusion has been discussed in the literature for at least half a century [13]; Wilburn, Blaufuss, and Bennett, as far back as 1975 [13], reported the occurrence of this adverse event in two of the 13 participants from their prospective cohort study. The authors' study primarily aimed to determine the effectiveness of minoxidil in persons with hypertension [13].

Pericardial effusion constituted 1.27% of the 2747 reports. The incidence of pericardial effusion with the use of oral minoxidil has been reported to be 3% [14, 15, 16]. Though spontaneously reported data are not designed to presume incidence [17], we infer that a high majority of minoxidil‐related pericardial effusions was captured in the FAERS. Moreover, we found that, across 97% of reports for pericardial effusion and oral minoxidil, this adverse event was reported by either a physician, pharmacist, or a health professional. While pericardial effusion is the focal point of the current paper, discussions thereof—in the literature—have not been without mention of “pericardial tamponade,” [14, 15] a potentially life‐threatening consequence of pericardial effusion. The reporting rate of 1.27% coupled with almost all minoxidil‐related pericardial effusions being reported by the healthcare workforce could serve as a telltale sign that the medical system has been providing decent care insofar as detection of this adverse event; moreover, the discourse around pericardial effusion and oral minoxidil is no stranger to the medical literature—and hence to medical practitioners.

Discussion of this relationship has also been as recent as this year: Kincaid et al. [18] investigated whether there is a positive correlation between low‐dose oral minoxidil (LDOM) and pericardial effusion in persons with alopecia; participants were consecutively sampled between January and April 2023 at a dermatology clinic. The authors found that the prevalence of pericardial effusion did not significantly differ between LDOM users (*n* = 51) and nonusers (*n* = 49) who had been diagnosed with alopecia [18].

Findings from Wilburn, Blaufuss, and Bennett [13], Kincaid et al. [18], and many others [5, 14, 15, 19, 20, 21] collectively spotlight the literature's exiguity of conclusive evidence, because the question of whether oral minoxidil causes pericardial effusion is far from being answered—despite being asked at least five decades ago.

The current study attempted to provide more understanding of oral minoxidil's etiological role in the development of pericardial effusion by examining spontaneously reported data from the FAERS across an observation period of 18.5 years (i.e., 1st quarter of 2006 to the 2nd quarter of 2024 inclusive). We identified 2747 reports for oral minoxidil with complete information on the total daily dose of up to 100 mg daily. Across the 2747 reports, the most common indication for minoxidil use was hypertension, which resonates with the fact that participants of studies on oral minoxidil are often hypertensive [13, 15].

Also, within the past five decades, Reichgott; Akinleye, Jamil, and Dogbey [15, 20], and others have suggested that the relationship between oral minoxidil and pericardial effusion is best described as a dose‐independent drug reaction, as the occurrence of this AE is unpredictable and dose independent. Our findings support this suggestion [15, 20]; our results support that there is no correlation between dose and occurrence of pericardial effusion.

Like Kincaid et al.'s [18] study, the results of the current study showed no relationship between age and the occurrence of minoxidil‐induced pericardial effusion. Furthermore, the nonsignificance we found between the occurrence of this adverse event and age, weight, and dose reinstate the literature's suggestion of a dose‐independent relationship between pericardial effusion and the vasodilator. In the current dataset, no pericardial effusions were recorded when the minoxidil dose was less than 2.5 mg/day. There were 2/35 reports at 2.5 mg/day and 8/35 reports at 5 mg/day (Table 1).

There are six reports (4 males, 2 females) (United States 4, United Kingdom 1, Switzerland 1) of pericardial effusion with topical minoxidil therapy (Table 3). The age range was 32–65 years. The six reports were well below 1% of all the reports recorded for AEs for all formulations of minoxidil. In four of these six cases, there was a positive dechallenge; in one case, there was a negative rechallenge. Although the causal relationship between the topical application of minoxidil and pericardial effusion is not proven, one can speculate that there may be enhanced absorption of topically applied minoxidil in some cases, and in others, there may be enhanced susceptibility to the effects of topical minoxidil. Additionally, a small percentage (1.4%) of topically applied minoxidil is absorbed through a normal scalp, supporting the idea that the potential development of PEs in some patients may be independent of the minoxidil dose [22]. In clinical practice, some patients have experienced headaches, palpitations, and a sense of fainting following the application of topical minoxidil.

**TABLE 3:** Characteristics of Reports for pericardial effusion and use of topical minoxidil across 1st quarter of 2006 to 2nd quarter of 2024 in the United States Food and Drug Administration Adverse Event Reporting System (FAERS).

ID	Sex	Drug	Country which reported the AE	Age, years	Dechallenge	Rechallenge	Reporter's occupation
1	Male	Minoxidil extra strength (for men)	United States	32	Positive dechallenge		Other health Professional
2	Male	Regaine extra strength‐mens (5%)	United Kingdom			Unknown	Consumer
3	Female	Regaine	Switzerland	40	Positive dechallenge	Negative rechallenge	Physician
4	Female	Men's rogaine unscented formula	United States			Unknown	Physician
5	Male	Minoxidil topical solution	United States	43	Positive dechallenge		Physician
6	Male	Minoxidil topical solution	United States	65	Positive dechallenge		Physician

Pericardial effusions may be asymptomatic when reported with oral minoxidil use [18]. When pericardial effusions become symptomatic, dyspnea, chest tightness, chest pain, dizziness, palpitations, nausea, and peripheral edema may develop. In more severe instances, cardiac tamponade can occur. Patients should be counseled about the possible development of the above adverse events. Patients with pre‐existing cardiac and renal disease should not be prescribed minoxidil without specialist consultation and follow‐up.

Notwithstanding the limitations [23] of spontaneously reported data, the statistically significant findings that the current study found for dechallenge and purported role arguably give reason(s) to ascertain a causal role in oral minoxidil's etiology for pericardial effusion. The findings of the current study support the conduct of future related prospective cohort studies—and the inferences therefrom can reiterate the findings of the current study.

## 5 Conclusion

Although the reporting frequency of pericardial effusion is relatively low compared to other side effects, our study identifies its occurrence with minoxidil use in the FAERS database. Also, it may not be dose dependent. Before prescribing oral minoxidil for androgenetic alopecia, clinicians should conduct a thorough medical history and physical examination to identify pre‐existing conditions that could increase the risk of pericardial effusion. Baseline cardiac evaluation should be considered, particularly for patients with a history of cardiovascular disease. Regular follow‐ups are recommended to monitor for symptoms and signs such as shortness of breath, chest tightness, chest pain, palpitations, dizziness, nausea, and peripheral swelling. Patients should be counseled on the potential for these adverse events. Additionally, minoxidil should be avoided in patients with pre‐existing cardiac or renal disease without specialist consultation and close monitoring.

## Author Contributions

Conception of the manuscript was done by M.T. and A.K.G. Data analysis was performed by M.A.B. The manuscript was drafted by M.A.B., A.K.G., and M.T., and substantively edited and revised by R.H., G.W., V.P., A.K.G., and M.T.

## Disclosure

V.P. has received grants from AbbVie, Bausch Health, Celgene, Eli Lilly, Incyte, Janssen, LEO Pharma, L'Oréal, Novartis, Organon, Pfizer, Sandoz, and Sanofi, received payment or honoraria for speaking engagement from Sanofi China, participated on an advisory board for LEO Pharma, Novartis, Sanofi, and Union Therapeutics, and received equipment donation from L'Oréal. V.P. declares that the interests do not affect the objectivity or integrity of this article.

## Ethics Statement

Approval from an ethics board was not required as there was no direct involvement with human participants.

## Conflicts of Interest

The authors declare no conflicts of interest.

## Data Availability

The data that support the findings of this study are available from the corresponding author upon reasonable request.
